# Impact of Climate Change on the Narrow Endemic Herb *Psilopeganum sinense* (Rutaceae) in China

**DOI:** 10.1002/ece3.71042

**Published:** 2025-02-28

**Authors:** Ruixiong Deng, Kaitong Xiao, Xin Chen, Beibei Huang, Haoran Li, Lin Wu, Hang Ning, Hui Chen

**Affiliations:** ^1^ Hubei Key Laboratory of Biological Resources Protection and Utilization (Hubei Minzu University) Enshi Hubei China; ^2^ College of Forestry and Horticulture Hubei Minzu University Enshi Hubei China; ^3^ State Key Laboratory for Conservation and Utilization of Subtropical Agro‐Bioresources, Guangdong Key Laboratory for Innovative Development and Utilization of Forest Plant Germplasm, College of Forestry and Landscape Architecture South China Agricultural University Guangzhou China

**Keywords:** climate change, Psilopeganum sinense, random forest algorithm, suitable habitats

## Abstract

*Psilopeganum sinense* is a perennial herb endemic to the Three Gorges Reservoir Area (TGRA) of the Yangtze River and its surrounding regions. This species is crucial for ecological conservation and regional socioeconomic development. Recent extreme weather events in the TGRA have directly and indirectly caused local losses of numerous wild populations of *P. sinense* . Given the severe survival crisis induced by climate change, it is essential to explore the effects of climate change on the potential distribution of *P. sinense* . Although there is a general awareness of the adverse effects of climate change on various species, there is a lack of comprehensive studies focusing on the long‐term effects and detailed climatic variables influencing the distribution of *P. sinense* . In this study, we aimed to use the random forest (RF) algorithm to analyze the redistribution of *P. sinense* across several critical climatic periods. The results indicated that the main variables limiting the present geographical distribution of *P. sinense* were precipitation seasonality and the mean diurnal range. Currently, *P. sinense* is mainly distributed in the riparian zone of the TGRA and its surrounding areas, exhibiting a relatively narrow climatic niche and habitat fragmentation pattern. Historically, distributions under past climatic conditions were relatively intact and more extensive than the current distribution area. During the last interglacial period, a broad distribution of highly suitable areas was observed in eastern Sichuan Province, northern Chongqing, and central Hubei Province, exhibiting a continuous distribution pattern. Future climate scenarios indicated a projected 32.84% decrease in suitable areas under RCP4.5–2050s. In northern Chongqing, the ecological corridors established in highly suitable habitats would fragment and gradually separate. Some previously unsuitable areas for *P. sinense* could transform into potentially suitable habitats because of climate change; however, these suitable areas might exhibit fragmented and discrete distribution patterns. In general, both the shrinkage of suitable habitats and habitat fragmentation would compress the already limited survival space of *P. sinense*, leading some populations to prematurely confront critical survival decisions under severe climate pressures. Our results not only provide a scientific basis for managing *P. sinense* resources in the context of climate change but also serve as an important reference for restoring wild *P. sinense* populations.

## Graphical abstract

Psilopeganum sinense is a perennial herb endemic to the Three Gorges Reservoir Area (TGRA) of the Yangtze River and its surrounding regions. This species is crucial for ecological conservation and regional socioeconomic development. However, the indeed fact that the TGRA has experienced significant extreme weather events in recent years has directly and indirectly caused the local loss of many wild populations of *P. sinense* . Therefore, our results can not only provide a scientific basis for the management of *P. sinense* resources in the context of climate change, but also provide an important reference for the restoration of wild populations.

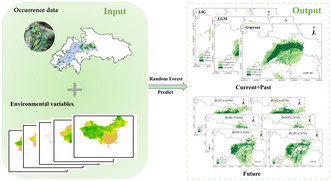

## 1 Introduction

Global warming has emerged as a major challenge linked to climate change over the past century. The Intergovernmental Panel on Climate Change Fifth Assessment Report indicates that the global average temperature has increased by approximately 0.85°C over the past 130 years (Pachauri 2016). Correspondingly, surface temperatures in China have increased considerably since 1900, exhibiting an average annual warming rate of approximately 0.08°C/10 years (Ren, Ding, et al. 2012). The persistence of elevated temperatures in the future appears inevitable. Climate change may result in three possible outcomes for different species: persistence through migration, adaptation to new conditions, or local extinction (Atkins and Travis 2010). Assessing the vulnerability of plant species to rapidly changing climates involves using species distribution models (SDMs) (Austin 2007). These models can be used to predict species climate niches and project their potential future range shifts. SDM results can guide the development of adaptive management strategies, including assisted migration, to mitigate the effects of climate change on biodiversity (Wang et al. 2012).

Rare and narrow‐ranging species are of considerable concern within the scientific community and among institutions tasked with achieving conservation targets and evaluating progress toward ambitious conservation goals. Mouillot et al. (2013) have demonstrated that rare species are not functionally redundant within ecosystems; instead, they possess unique traits that can have disproportionate ecosystem‐level effects if they are to become extinct. Gabriele et al. (2014) have indicated that range‐restricted plant species would lose approximately 50% of their current range by 2020, based on an analysis of the potential effects of climate change on 22 plant species restricted to the central‐northern Mediterranean region. Loarie et al. (2008) have reported that as much as 66% of the endemic plant taxa in California are set to experience > 80% range reduction within the next century.

In China, numerous scholars have identified that wild plant species with extremely small populations are facing severe threats from predicted climate change in the 21st century (Ren, Zhang, et al. 2012; Yang et al. 2020). Moreover, an increasing number of their habitats are being lost (Zhang et al. 2021; Oin et al. 2022). Therefore, accurate predictions are essential for alerting scientists and decision‐makers to potential future risks, enhancing the attribution of biological changes to climate change, and supporting the development of proactive strategies to mitigate the impacts of climate change on geographical distribution ranges (Pereira et al. 2010; Parmesan et al. 2011). Estimating the future potential range of a species, although not directly addressing the causes of extinction, indicates that a substantial reduction in potential distribution is likely to increase the risk of local extinction (Thomas et al. 2004; Thuiller et al. 2005).

The Three Gorges Reservoir Area (TGRA) is located in the central region of the Yangtze River Basin, China, and represents a typical ecotone. An ecotone is a transitional zone between two ecosystems, exemplified by farming–pastoral zones, water–l and junctions, forest edges, and desert margins. Ecotones are characterized by ecosystems exhibiting low resilience to disturbances, high susceptibility to degradation, and considerable challenges in restoration (Lind et al. 2019; Zhao et al. 2023). The TGRA currently faces substantial climate‐change challenges. Over the past 60 years, the TGRA has exhibited an evident warming trend attributable to global warming, with the most pronounced increases occurring during winter. In recent years, high‐temperature events have become more frequent during the summer. Wu et al. (2023) noted an increasing trend in temperature in the TGRA, particularly upstream, with temperature increases concentrated between 0.3°C and 0.5°C/10a. Seasonally, the increasing temperature trend was most pronounced during spring. Except for autumn, relative humidity in most regions in the TGRA showed notable downward trends, signifying an overall drying pattern. Total precipitation exhibited minimal change, with the most substantial variation observed during summer. Zeng et al. (2019) suggested that climate change in the TGRA was primarily influenced by environmental climate change. The climate change assessment of the TGRA indicates that the turning point for annual mean temperature and annual mean relative humidity trends occurred around 1996 for the 1973–2013 period. Conversely, annual precipitation showed no evident turning point (Zhang, Liu, et al. 2022). Therefore, the flora and fauna inhabiting the TGRA may be experiencing the effects of this climate change. Therefore, an urgent need exists to formulate effective conservation measures for specific species in the context of climate change.

*Psilopeganum sinense*, a perennial herb endemic to China and classified as a rare and endangered second‐grade state‐protected species, is primarily distributed in the TGRA (Tang et al. 2014). Meanwhile, *P. sinense* is a monotypic genus that plays an important role in ecological conservation (Appelhans et al. 2016). However, *P. sinense* has been extensively overharvested by local residents because of its widespread use as a traditional medicinal herb and spice, posing severe threats to the survival of its populations (Huang 2001). Field surveys have identified numerous natural populations of *P. sinense* located in the riparian zone of the TGRA. *P. sinense* is a typical representative of species distributed in the riparian zone (Wu et al. 2023). The riparian zone of the TGRA represents a typical ecotone characterized by periodic submersion or exposure to water resulting from the storage or discharge of water from the reservoir. The riparian zone of the TGRA spans 5578 km in length, features a vertical drop of 30 m, and encompasses a total area of 349 km^2^. It extends from Jiangjin District in Chongqing to Yichang City in Hubei Province, fluctuating between a high‐water level (175 m) during winter and spring and a low‐water level (145 m) during summer and autumn (Li et al. 2023). The original vegetation habitat in the riparian zone has experienced substantial changes due to long‐term repeated flooding exposure and dry‐wet cycle alternation. Numerous indigenous species are ill‐suited to the altered environment, resulting in survival challenges that contribute to the gradual decline of vegetation and the permanent loss of the original habitats (Breton et al. 2023). Therefore, the water storage methods in the TGRA may have altered the hydrological climate and ecological environment of this region, posing a potential survival crisis for *P. sinense* . Therefore, it is crucial to explore the potential distribution of *P. sinense* in the context of climate change.

To effectively address the challenge of climate change, the response of the indigenous resident *P. sinense* to climate change in the TGRA should be projected. This approach facilitates a timely understanding of the risks posed by climate change to vegetation in the TGRA and establishes a scientific basis for formulating adaptive management strategies. Therefore, in this study, we aimed to use random forest (RF) to model the current, past, and future potential distributions of *P. sinense* . This research had three objectives: (1) to identify the dominant environmental variables influencing the distribution range of *P. sinense* populations; (2) to project future distributions of *P. sinense* populations under various climate change scenarios; and (3) to develop effective protection strategies for indigenous residents of *P. sinense* in the TGRA based on these projections.

## 2 Materials and Methods

### 2.1 Study Area

We used a proxy study area that covered the natural distribution of *P. sinense* , where populations with fragmented distributions inhabited the riparian zone of the TGRA and its surrounding environment. The TGRA exhibits a subtropical monsoon climate characterized by predominantly evergreen broad‐leaved forests, with an average annual temperature ranging from 14.9°C to 18.5°C and average annual precipitation between 1000 and 1300 mm (Song et al. 2017). Summer precipitation demonstrates considerable spatial heterogeneity: some areas experience heavy rainfall, whereas others receive minimal precipitation. Rainstorms occur more frequently in this region, with large intra‐ and inter‐annual variations in precipitation. Consequently, heavy precipitation is likely to result in regional flooding and secondary disasters, including collapses, landslides, and mudslides, thereby rendering the ecology of the region very fragile (Wang et al. 2024).

### 2.2 *P. sinense* Presence Records

In this study, we assembled a database of the presence of *P. sinense* using three methods. First, we conducted field investigations from 2022 to 2024 with support from the Department of Forestry Protection. The county forestry departments were regarded as survey units based on the known distribution areas of *P. sinense* . Along the TGRA, the fieldwork commenced in Jiangjin District, Chongqing Municipality, and concluded in Yiling District, Yichang City, Hubei Province, China. A total of 26 counties or districts were investigated, including Jiangjin, Jiulongpo, Dadukou, Banan, Shapingba, Naan, Beibei, Yuzhong, Jiangbei, Yubei, Changshou, Fuling, Wulong, Fengdu, Zhongxian, Shizhu, Wanzhou, Kaizhou, Yunyang, Fengjie, Wuxi, Wushan, Badong, Zigui, Xingshan, and Yiling. Subsequently, we conducted investigations in the counties adjacent to the TGRA. A total of 9 presence sites of *P. sinense* were identified (Figure S1). The distance between the sampling sites was > 20 km. When the distance between two or more presence points was < 20 km, only one presence point was recorded. The specific information recorded for each point included the location name, longitude, latitude, and altitude. Second, we gathered presence data from various online sources, such as the Global Biodiversity Information Facility (GBIF, http://www.gbif.org), Chinese Virtual Herbarium (CVH, http://www.cvh.org.cn/), China National Forest Resource Continuous Investigation Platform (http://www.stgz.org.cn/), China's Nature Reserve Specimen Resource Sharing Platform (http://www.papc.cn/), and China's Teaching Specimen Resource Sharing Platform (http://mnh.scu.edu.cn/). Third, we acquired presence records from relevant literature sources (Song et al. 2004; Yang et al. 2007; Tang et al. 2014). After excluding data with incomplete geographic information or minimal coordinate differences, we compiled 85 presence records of *P. sinense* .

**FIGURE 1:**
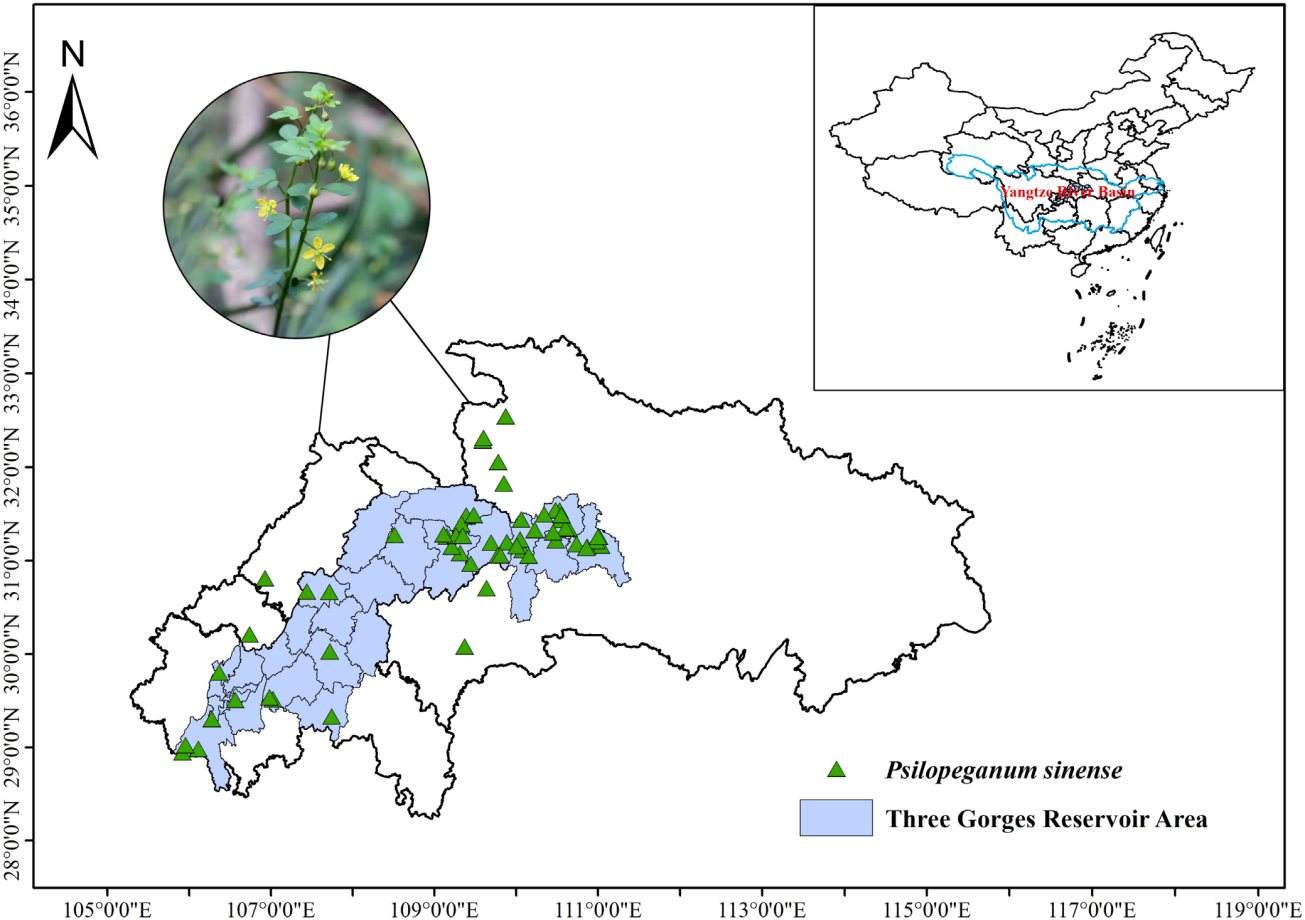
Current distribution of *P. sinense* in China.

However, given the limited reference data available in relevant literature and on websites, field sampling remained the primary method for obtaining data on *P. sinense* . Moreover, the TGRA is a unique geographical region in China, with certain areas restricted from entry in accordance with national policies and laws. Therefore, sampling bias inevitably affects species distribution modeling. The sampling intensity often differs between sites, and sampling sites are typically biased toward areas that are more accessible. Oversampling in some geographic spaces can lead to redundancy in the ecological space, thereby affecting the simulation of the ecological needs of a species during model construction. To counter the sampling bias, we used ArcGIS 10.8 to randomly generate 300 “pseudo‐absence” points as background points. Next, we selected points for model calibration through a subsampling regime aimed at minimizing the sampling bias and spatial autocorrelation. We generated models using all the occurrence points and measured the spatial autocorrelation among the model pseudo‐residuals by calculating Moran's I across multiple distance classes. Then, the significance was determined through permutation tests, and the occurrence point nearest to the centroid of each grid cell was selected. The procedure greatly reduced the sampling bias and spatial autocorrelation, resulting in a uniform distribution of occurrence points across geographic space. This filtering process resulted in a final dataset comprising 59 presence records for *P. sinense* (Figure 1). The coordinates of the 59 presence records of *P. sinense* are shown in Table S1.

### 2.3 Environmental Data

We obtained environmental data consisting of 19 bioclimatic variables from WorldClim at a resolution of 30 arc‐seconds (Table S2). These variables encompassed the past, present, and future periods, all of which were calibrated and standardized. A national map derived from the National Fundamental Geographic Information System served as the analytical base map (http://www.ngcc.cn). For past periods, we used data from the last interglacial (LIG) and last glacial maximum (LGM) periods. These data were sourced from the Coupled Model Intercomparison Project and reconstructed using the CCSM4 model. Future scenarios for the 2050s and 2070s were derived from the downscaled and bias‐corrected global climate models provided by WorldClim. These scenarios utilized data from the BCC‐CSM1.1 model developed by the Beijing Climate Center, a global climate model released by the Intergovernmental Panel on Climate Change in 2013. The Fifth Intergovernmental Panel on Climate Change report presented four Representative Concentration Pathways, which illustrate varying levels of greenhouse gas emission mitigation: RCP2.6 (stringent), RCP4.5 (moderate), RCP6.0 (moderate), and RCP8.5 (high) (Rogelj et al. 2018). In this study, RCP 2.6, RCP 4.5, and RCP 8.5 were selected to project the future distributions of *P. sinense* in the 2050s and 2070s.

To address the potential overfitting arising from multicollinearity among environmental factors, we employed two filtering methods. Initially, we used Pearson's correlation coefficients (r) to assess the correlations among environmental variables, selecting only one variable (*r* > 0.8) from each set of strongly correlated factors. Subsequently, we implemented the recursive feature elimination (RFE) technique to further refine the remaining variables. RFE is a robust feature selection method that iteratively constructs models and eliminates the least important features at each step (Chen et al. 2018). This approach identifies the most influential subset of features that significantly contribute to the predictive performance of the model. Different feature subsets were evaluated through cross‐validation to ensure the robust generalization of unseen data. The iterative refinement process continues until a predefined number of features or performance threshold for the model is reached, resulting in an optimal feature set and a comprehensive assessment of model performance. This iterative approach complements the initial correlation analysis, facilitating the selection of the most informative variables while mitigating overfitting and enhancing model accuracy.

### 2.4 Construction and Validation of the RF Model

In this study, the RF algorithm from the BioMod2 package in R was used to predict the potential distribution of *P. sinense* (Thuiller et al. 2023). RF functions by employing decision trees and incorporating random attribute selection via bagging technology during the training phase. This methodology generates multiple classification and regression trees that are subsequently subjected to regression analysis on selected subsamples. Consequently, RF effectively mitigates the issue of overfitting, which is common in many other species distribution models, leading to more accurate and stable predictions (Breiman, 2001). In addition, RF demonstrates proficiency in managing extensive datasets, addressing missing data, tackling nonlinear and collinearity challenges, and assessing variable significance directly (Bi et al., 2013; Bradter et al., 2013). By integrating the pseudo‐presence points randomly generated by ArcGIS 10.8, 300 presence and pseudo‐presence datasets were established as a point‐based input dataset. Subsequently, 10‐fold cross‐validation was used to train and validate the model. Of the 10 subsets, a single subset was retained as validation data for testing the model, whereas the remaining subsets were used as training data. For each subset, 70% of the occurrence data were used to train the single model, whereas the remaining data were used to test the predictive performance of the model. The model was then run 10 times for cross‐validation, yielding more accurate and realistic predictions.

The statistical accuracy of the model was assessed by the area under the curve (AUC), true skill statistic (TSS), and kappa values (Table S3) (Allouche et al. 2006). The AUC is defined as the area enclosed by the receiver operating characteristic (ROC) curve and coordinate axis. Metrics assessed using the ROC curve often range from 0 to 1, with elevated values indicating greater model accuracy (Wiley et al. 2003). The Kappa coefficient, which ranges from −1 to 1, serves as a statistical index for measuring classification accuracy. The TSS, an enhanced test index derived from the Kappa coefficient, primarily converts continuous predicted values into binary values, with its range corresponding to that of the kappa coefficient (Hipólito et al. 2015). A value closer to 1 indicates a higher model accuracy.

### 2.5 Data Analyses of Key Bioclimatic Variables and Habitat Suitability

The weights of the environmental variables affecting the distribution of *P. sinense* were established using rigorous statistical analyses. Variables with a cumulative contribution rate exceeding 80%, ranked in descending order of importance, were identified as key factors. Ultimately, a more precise and realistic distribution of *P. sinense* was determined.

The output TIF format layers were imported into ArcGIS 10.8 for further analysis, and a final distribution map was generated. Each raster value in the species distribution layer represents the probability of the species occurring in that area and ranges from 0 to 1. However, no standardized criteria for classifying habitat suitability levels for species currently exist (Liu et al. 2005). The habitat suitability index (HSI) was used to classify the suitable distribution of *P. sinense* . This method was first proposed by the United States Fish and Wildlife Service in the Habitat Assessment Program, and the HSI is widely used to quantify the relationship between habitat preference and habitat factors of an organism (Wakeley 1988; Roloff and Kernohan 1999; Lu et al. 2012; Xue et al. 2016). Therefore, the potential distribution of *P. sinense* was divided into four categories: unsuitable, marginally suitable, moderately suitable, and highly suitable. Then, the product of the number of grid cells and their spatial resolution was employed to calculate the area suitable for the current, past, and future climate scenarios.

## 3 Results

### 3.1 Screening of Important Environmental Variables and Model Performance

**FIGURE 2:**
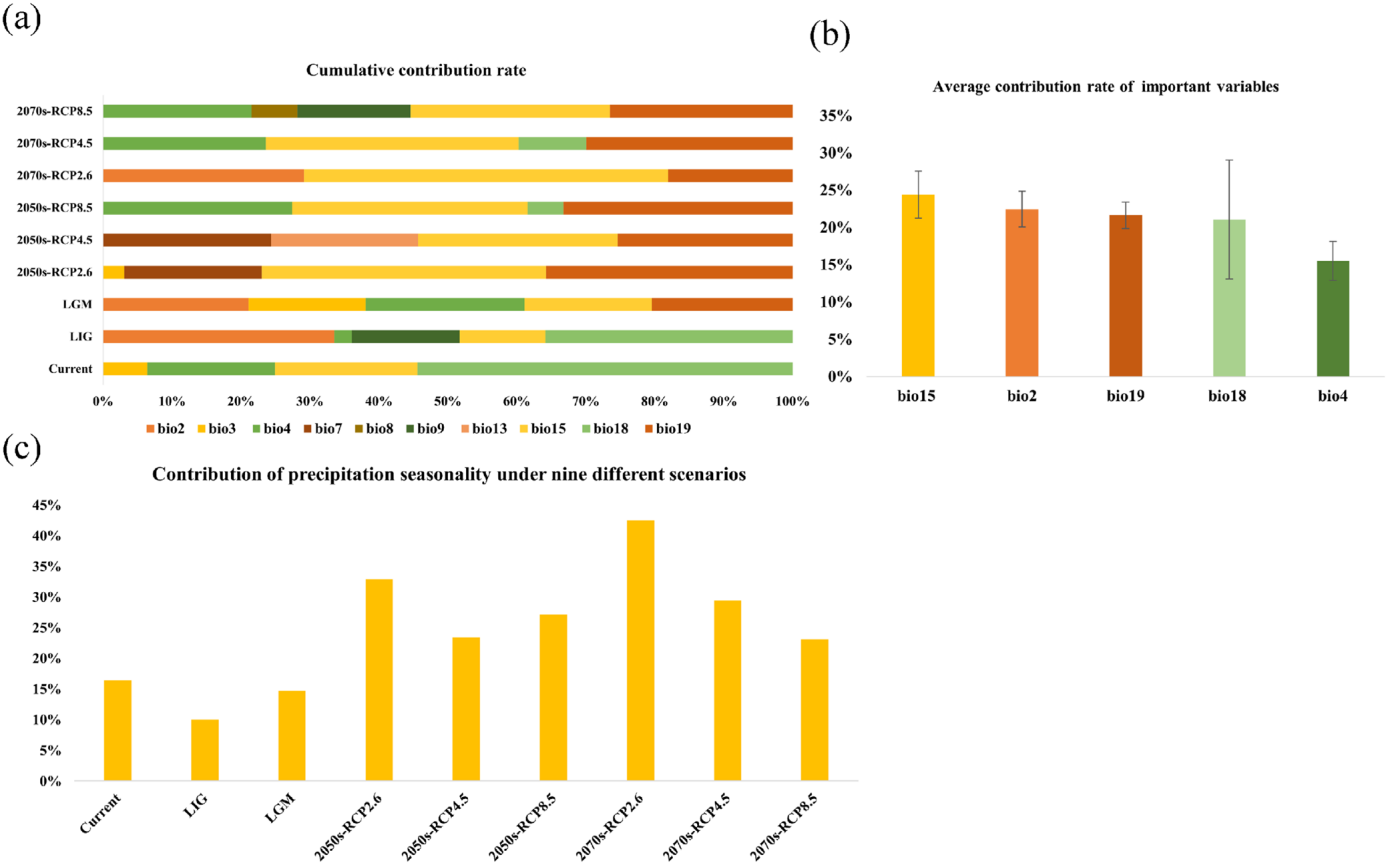
Sorting and filtering of important variables. (a) Cumulative contribution rate. (b) Average contribution rate of important variables. (c) Contribution of precipitation seasonality under nine different scenarios.

Based on the RF output results, we identified the key environmental variables affecting the geographical distribution of *P. sinense* (Figure 2). Five bioclimatic variables accounting for the top 80% of the cumulative contribution rate were obtained for each model (Figure 2a). The most important environmental variables with the top five average contribution rates were as follows: precipitation seasonality (Bio15, 24.3%), mean diurnal range (Bio2, 22.4%), precipitation of the coldest quarter (Bio19, 21.6%), precipitation of the warmest quarter (Bio18, 21.1%) and temperature seasonality (Bio4, 15.5%) (Figure 2b).

The contribution of precipitation seasonality was the highest among the five key bioclimatic variables. Therefore, it can be regarded as the most important bioclimatic variable. Its contributions to each of the nine climate scenarios are shown in Figure 2c. In addition, the cumulative contribution rate of the five variables revealed that precipitation factors had a higher total cumulative contribution to the distribution of *P. sinense* than temperature factors, indicating a greater sensitivity of *P. sinense* to precipitation than to temperature.

**FIGURE 3:**
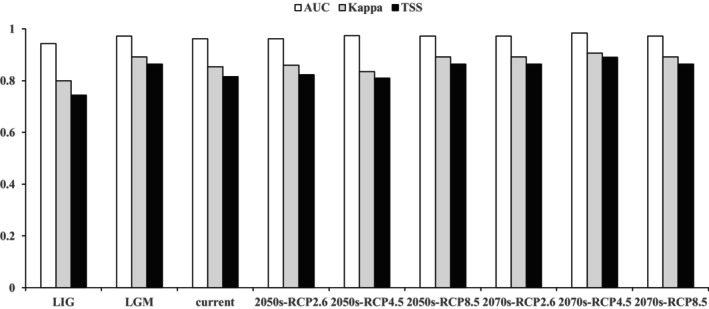
Three indicators for predicting the accuracy of current, past, and future climate scenarios for *P. sinense*.

The AUC, Kappa, and TSS values for each period derived from the model‐based predictions are presented in Figure 3. The results showed that the model had high reliability, with AUC values exceeding 0.9, Kappa values exceeding 0.8, and TSS values exceeding 0.8 for each period. This suggests that the model exhibited strong generalization and excellent predictive performance.

### 3.2 Suitable Distribution of *P. sinense* From the Past to the Future

#### 3.2.1 Suitable Distribution of *P. sinense* Under Current Climate Conditions

**FIGURE 4:**
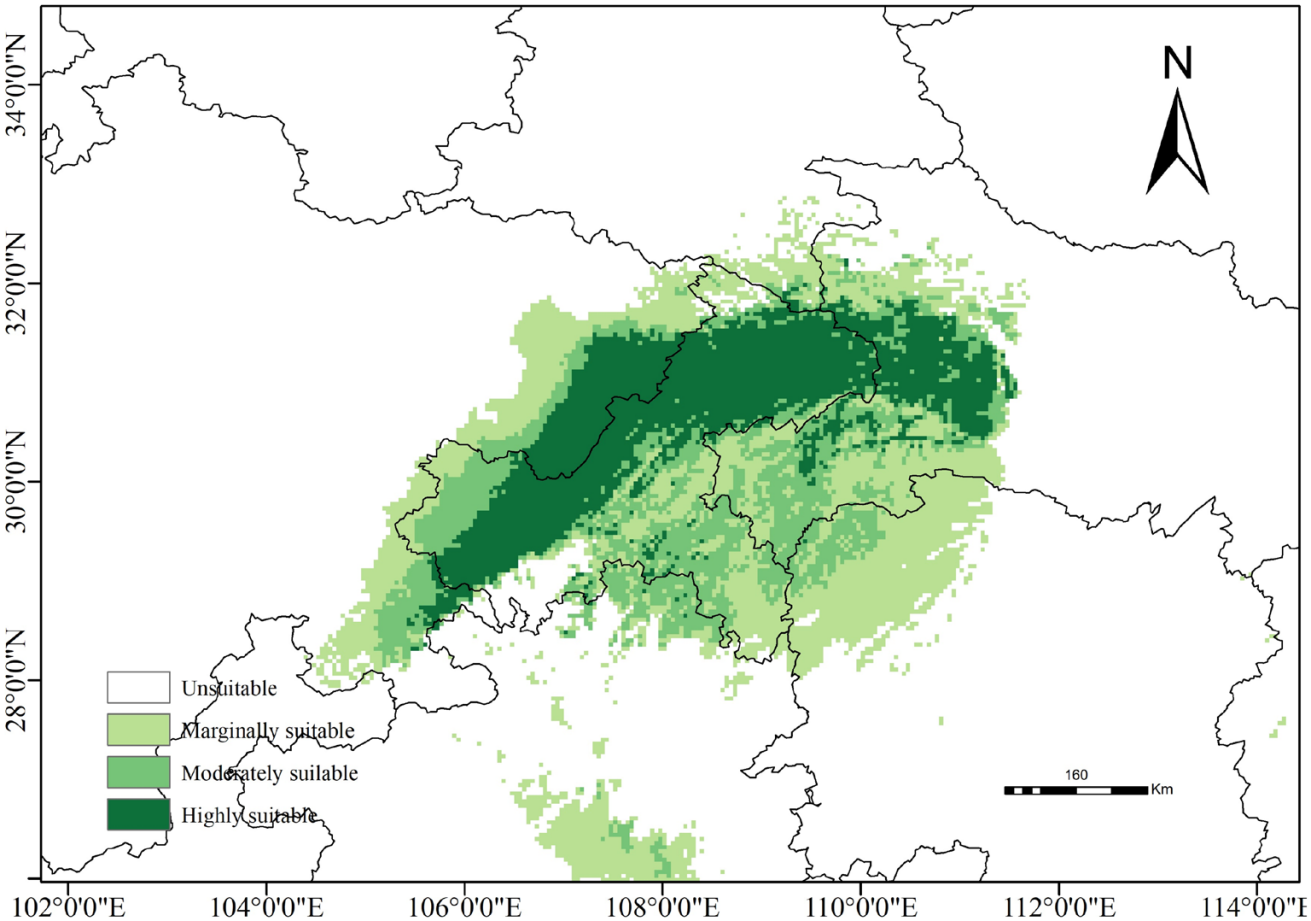
Potential distribution of *P. sinense* in China under current climate scenarios.

**FIGURE 5:**
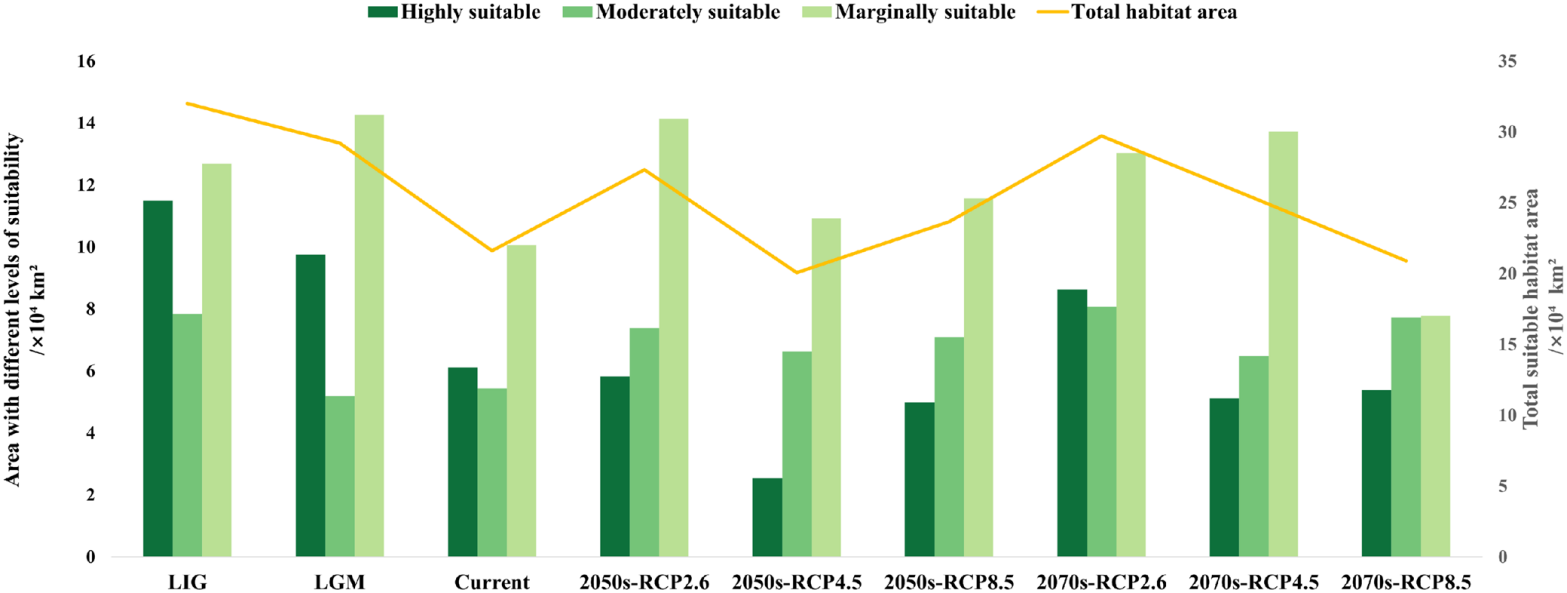
The total suitable area of *P. sinense* under different climate scenarios and the suitable area under various categories.

The projected current potential distribution of *P. sinense* is shown in Figure 4. Suitable habitats are predominantly concentrated in the TGRA; the junction region of Chongqing, Guizhou, Hunan, and Hubei Provinces; and the junction area of Sichuan, Shaanxi, and Hubei Provinces. These habitats had a total area of approximately 21.62 × 10^4^ km^2^. The highly, moderately, and marginally suitable areas were 6.11, 5.44, and 10.07 × 10^4^ km^2^, respectively. Among these, highly suitable habitats in the TGRA exhibited a dominant distribution range (Figure 5).

#### 3.2.2 Suitable Distribution of *P. sinense* Under Historical Climate Conditions

**FIGURE 6:**
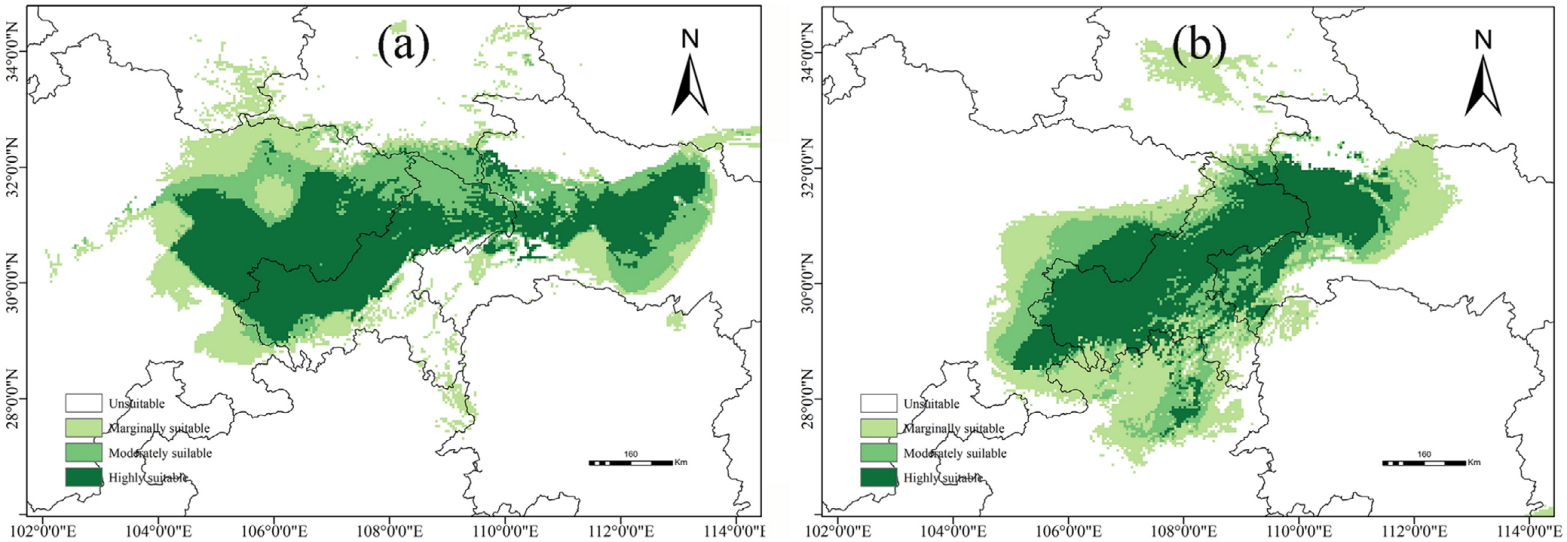
Potential distribution of *P. sinense* in China under past climate scenarios. (a) in the LIG; (b) in the LGM.

The suitable distribution of *P. sinense* under the two historical climatic conditions is shown in Figure 6. During the LIG period (Figure 6a), there was a broad distribution of highly suitable areas in eastern Sichuan Province, northern Chongqing, and central Hubei Province, with a continuous distribution pattern. Overall, the total area amounts to 32.01 × 10^4^ km^2^, of which the highly suitable area is ca. 11.49 × 10^4^ km^2^, and the moderately and marginally suitable areas are ca. 7.83 and 12.68 × 10^4^ km^2^, respectively.

During the LGM period (Figure 6b), the total suitable area was approximately 29.21 × 10^4^ km^2^. The highly, moderately, and marginally suitable areas were approximately 9.75, 5.19, and 14.27 × 10^4^ km^2^, respectively. Compared with the LIG period, the total suitable area decreased by 2.8 × 10^4^ km^2^, with the highly and moderately suitable areas decreasing by 1.74 and 2.65 × 10^4^ km^2^, respectively. However, the marginally suitable area increased by 1.59 × 10^4^ km^2^, indicating the potential emergence of numerous new suitable habitats in the future.

#### 3.2.3 Potential Distribution of *P. sinense* Under Future Climate Scenarios

**FIGURE 7:**
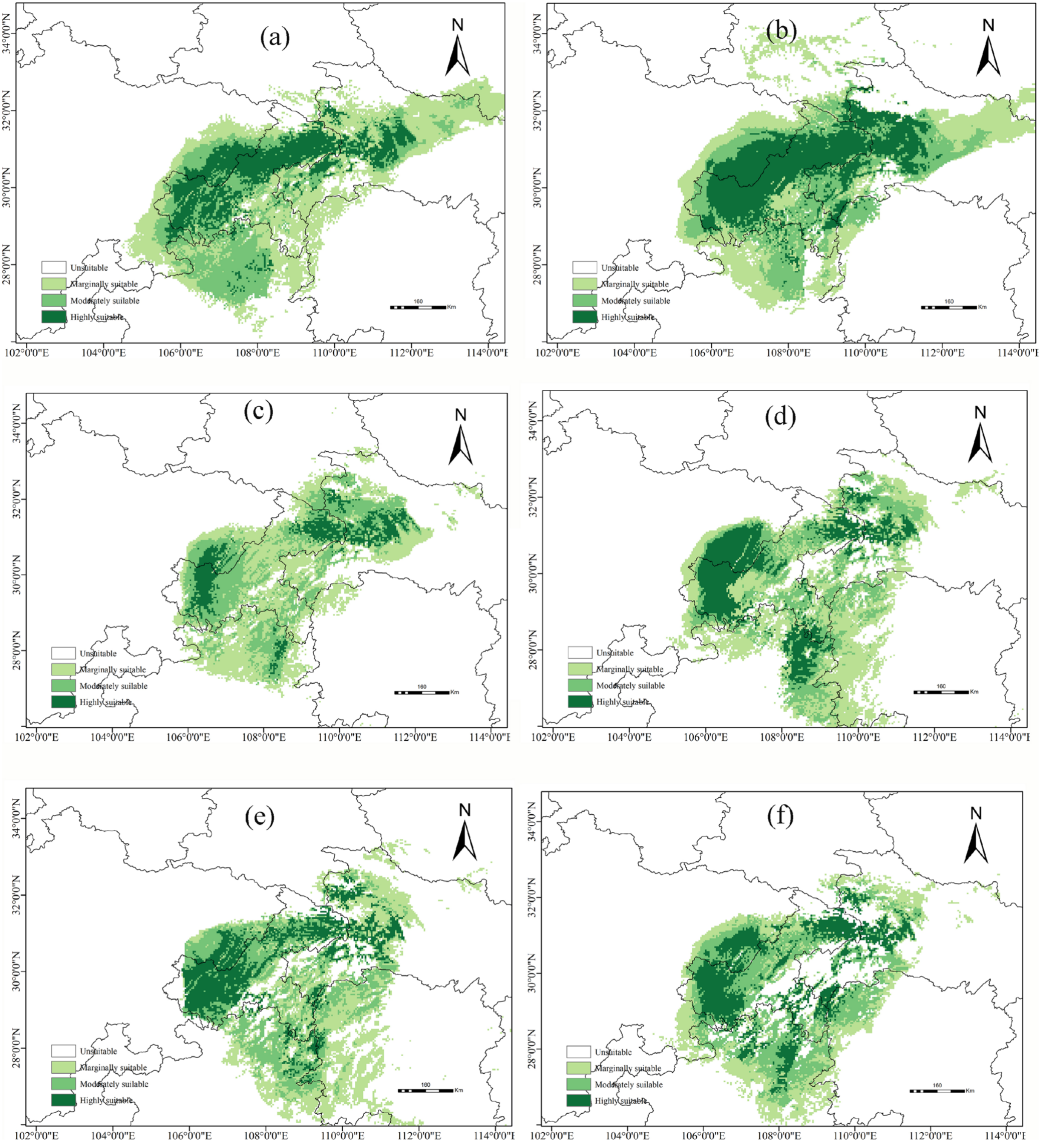
(a–f) Potential distribution of P. sinense in China under future climate scenarios. (a) RCP2.6–2050s, (b) RCP2.6–2070s, (c) RCP4.5–2050s, (d) RCP4.5–2070s, (e) RCP8.5–2050s, and (f) RCP8.5–2070s.

The future redistributions of *P. sinense* are presented in Figure 7. Under most future climate scenarios, the range of suitable habitats for *P. sinense* is projected to decrease. In northern Chongqing, ecological corridors established in highly suitable habitats are likely to fragment and become increasingly isolated. Some areas that were previously unsuitable for *P. sinense* may become potentially suitable habitats due to climate change. However, these suitable areas exhibit fragmented and discrete distribution patterns. Overall, both the shrinkage of suitable habitats and habitat fragmentation compress the limited survival space of *P. sinense* , leading some populations to confront critical survival decisions prematurely under extreme climate pressures.

Under the RCP2.6 scenario, the total suitable area for *P. sinense* exhibited an expanding trend, with its distribution profile resembling that of the current distribution (Figure 7a,b). The total suitable area for the 2050s was projected at 27.34 × 10^4^ km^2^. Among these, moderately and marginally suitable areas were predicted to increase by approximately 26% and 28%, respectively. The number of suitable habitats in northern Guizhou Province and northeastern Hubei Province increased significantly. By the 2070s, the total suitable area was projected to expand to a total of 29.73 × 10^4^ km^2^. In particular, the suitable habitat area in northern Guizhou Province exhibited an increasing trend, and there was a large area of suitable habitats for *P. sinense* populations to inhabit.

Under the RCP4.5 scenario, the suitable area was projected to be significantly reduced. The suitable habitat displayed evident fragmentation, mainly originating from the Hubei, Chongqing, and Guizhou Provinces (Figure 7c,d). In the 2050s, the total suitable area was projected at 20.07 × 10^4^ km^2^. However, the highly suitable area decreased the most, representing a 58% reduction compared with current climate conditions. The moderately and marginally suitable areas were approximately 6.63 and 10.91 × 10^4^ km^2^, respectively. By the 2070s, the highly, moderately, and marginally suitable areas were approximately 5.12, 6.49, and 13.73 × 10^4^ km^2^, respectively. Compared with the current distribution, both moderately and marginally suitable areas increased, whereas highly suitable areas decreased.

Under the RCP8.5 scenario, the suitable distribution of *P. sinense* exhibited a decreasing trend and fragmented pattern (Figure 7e,f). In the 2050s, the highly, moderately, and marginally suitable areas were projected at 4.98, 7.09, and 11.57 × 10^4^ km^2^, respectively. Compared with the current climate conditions, the highly suitable area decreased by 18.39%, whereas the moderately and marginally suitable areas increased by 23.30% and 13.03%, respectively. By the 2070s, the highly, moderately, and marginally suitable habitats were predicted to achieve 5.39, 7.73, and 7.78 × 10^4^ km^2^, respectively. Compared with current climatic conditions, the highly and marginally suitable areas decreased by 11.81% and 22.69%, respectively. Conversely, the moderately suitable area was projected to increase by 29.64%.

## 4 Discussion

The projected results indicate that the current distribution of *P. sinense* is approximately 21.58 × 10^4^ km^2^, primarily located in the TGRA. This distribution encompasses the junction region of the Chongqing, Guizhou, Hunan, and Hubei Provinces and the junction region of the Sichuan, Shaanxi, and Hubei Provinces. Field surveys and historical distribution records confirm that the predicted distribution range is consistent with the actual distribution area, providing further evidence of the high degree of accuracy of the model. Under past climatic conditions, the predicted historical distribution of *P. sinense* showed that the highly suitable range in the TGRA was consistent with the highly suitable distribution under current climatic conditions. During the LIG period, the north shore of the TGRA, especially northeastern Sichuan Province, contained numerous highly suitable areas, whereas only a few marginally suitable areas were present on the south shore of the TGRA. Another significant change during this period was the extension of the suitable area from the Sichuan Basin to the Jianghan Plain along the latitudinal line, forming an ecological corridor parallel to it. During the LGM period, the distribution range of *P. sinense* was consistent with its current distribution, with the suitability range predominantly along the TGRA. Significant increases were observed in the moderately and marginally suitable distributions on the southern shore of the TGRA compared with the LIG period. Overall, the distribution area under past climatic conditions was significantly larger than the current distribution area, indicating a gradual contraction of the distribution range of *P. sinense* toward the TGRA from the past to the present.

Under future climate scenarios, the highly suitable redistribution of *P. sinense* is projected to decrease, gradually presenting a more fragmented distribution pattern. Meanwhile, most of the areas experiencing changes in suitability are currently highly suitable areas, whereas the moderately and marginally suitable areas are expected to remain unchanged or even increase. Consequently, the original highly suitable regions are gradually being replaced by moderately and marginally suitable areas, resulting in the loss or disappearance of some suitable habitats. Overall, the suitable habitats of *P. sinense* have undergone and are expected to continue undergoing significant changes across past, present, or future timeframes. We hypothesize that these changes are likely driven by increased habitat fragmentation under future higher emission scenarios, which are characterized by highly irregular temperature and precipitation patterns, frequent extreme weather events, and substantial impacts on plant growth and propagation (Thomas et al. 2004; Monteith et al. 2015).

The results of variable screening revealed that precipitation and temperature considerably influenced the geographical distribution of *P. sinense* populations. In terms of precipitation, the dominant variables include precipitation seasonality, precipitation of the coldest quarter, and precipitation of the warmest quarter. Generally, *P. sinense* populations inhabit warmer and more humid areas in the TGRA and its surroundings (Tang et al. 2014). Notably, *P. sinense* is an ephemeral plant that requires special attention. At the macroscale, the geographical distribution pattern of the range‐restricted plant, *P. sinense* , is distinctly influenced by constraints imposed by climatic and geographical conditions (Zhang, Ye, et al. 2022). Typically, the climatic environment in this area is characterized by seasonal precipitation, with reduced precipitation in spring and winter and increased precipitation in summer and fall. Therefore, Bio15, Bio19, and Bio18 greatly influenced the geographical environment of *P. sinense* populations. These three variables were precisely selected, and precipitation seasonality might drive the growth and development of *P. sinense*. Ephemeral plants are opportunistic in nature. Therefore, their entire life cycle shows a high degree of dependence on precipitation, with *P. sinense* being no exception (Stromberg et al. 2009). In terms of temperature, the mean diurnal range and temperature seasonality were the dominant variables restricting the geographical distribution of *P. sinense* . Hydrothermal conditions collectively influence the geographical distribution of vegetation. These conditions strictly regulate each stage of the growth and development of ephemeral plants (Wang et al. 2022; Zeng et al. 2024). The interannual configuration of hydrothermal factors influences the geographic distribution patterns of *P. sinense* populations.

We explored the factors contributing to the endangered status of *P. sinense* . Two key findings of this study warrant careful consideration. On the one hand, *P. sinense* populations exhibited a narrow and fragmented geographical pattern. Yang et al. (2007) found that *P. sinense* exhibits low genetic diversity, and geographic isolation significantly affects population genetic differentiation. Studies have shown that low genetic diversity can lead to endangerment owing to reproductive barriers and difficulties in adapting to environmental changes (Pauls et al. 2013). The narrow and fragmented distribution pattern, along with a limited number of individuals, can trigger a chain of genetic consequences, such as inbreeding, which further reduces genetic diversity in *P. sinense* populations (Zhang and Ye 2011). On the other hand, the seeds of *P. sinense* exhibit unique characteristics that facilitate gravity dispersal. Its fruit is a follicle characterized by a depression at the top that resembles a mouth, which opens upon maturation. The seeds are approximately 1.5 mm in length and lack wings or thorns (Cheng 1997). Thus, the propagation mode of seeds greatly limits the long‐distance spread of local populations. Furthermore, low genetic diversity renders populations more susceptible to environmental selection pressures and diminishes their resilience to environmental changes, thereby hindering their adaptation to a wider climatic range and consequently elevating the risk of species extinction (Jump et al. 2009). Therefore, if the population size and natural regeneration cannot be effectively maintained, population degradation or even extinction of the entire population may occur.

Field surveys have revealed severe damage to the natural habitats of *P. sinense* . The construction of the Three Gorges Dam has further damaged its habitat, making it smaller. This habitat reduction has exacerbated the decrease in the number of *P. sinense* individuals, thereby leading to self‐incompatibility and lower gene flow (Morjan and Rieseberg 2004). Therefore, habitat fragmentation is a primary factor contributing to the decline in *P. sinense* populations. Climate change has further threatened the habitats of *P. sinense* populations, aggravating habitat fragmentation and potentially leading to the local extinction of some populations. These findings and our projected results highlight the urgent need for protection and management strategies for *P. sinense* populations. In this context, some researchers have implemented relocation measures to protect certain populations within the riparian zone of the TGRA (Song et al. 2004). However, these relocation protection efforts have been unsuccessful. For example, the *P. sinense* population relocated to the Wuhan Botanical Garden failed to produce new seedlings, and existing individuals continued to perish. Therefore, establishing relocation protection in areas that resemble original habitats is optimal because similar climatic conditions are more likely to ensure the survival of these populations.

Both relocation and onsite protection are essential for safeguarding as many local *P. sinense* populations as possible. Based on our predicted results, the network of nature reserves should be rationally zoned in accordance with the established protected areas in the TGRA and its surrounding environment. Current anthropogenic activities, including excessive reclamation and indiscriminate mining, directly contribute to the decline in *P. sinense* populations. Therefore, this behavior should be resolutely ceased to prevent further damage. First, immediate rescue and relocation protection measures should be implemented for *P. sinense* populations that suffer from artificial destruction and face severe threats. Second, the scientific value and practical significance of *P. sinense* should be vigorously publicized to raise societal awareness about the protection of these endangered resources. Protected areas should be established immediately to protect *P. sinense* in regions with abundant and flourishing populations. In addition, more investments should be made for further in‐depth research on *P. sinense* .

## 5 Conclusion

The RF model was used to project the potentially suitable distribution of *P. sinense* in China across various past, present, and future climate scenarios. The results showed that suitable habitats for *P. sinense* are mainly distributed in the TGRA and its surrounding areas. Key environmental variables, such as precipitation seasonality and the mean diurnal range, were identified as critical in limiting the current geographical distribution of *P. sinense* populations. Under past climatic conditions, historical distributions were relatively intact, with extensive highly suitable areas observed in eastern Sichuan Province, northern Chongqing, and central Hubei Province, forming a continuous distribution pattern. Projections under future climate scenarios suggest a decrease in the range of suitable habitats for *P. sinense* . Notably, in northern Chongqing, ecological corridors within highly suitable habitats are expected to fragment and gradually separate. In general, both the shrinkage of suitable habitats and habitat fragmentation will restrict the already limited survival space of *P. sinense* , potentially forcing some populations to confront critical survival decisions prematurely because of extreme climate pressures. Given these projections, strengthening conservation measures is essential. Based on the predicted results and prior research practices on the relocation protection of *P. sinense* , it is advisable to establish relocation protection in areas that closely resemble its original habitats. These areas with similar climatic conditions are more likely to ensure the survival of these populations. In conclusion, detailed protection and management strategies should be promptly developed and implemented. Immediate action is crucial.

## Author Contributions

**Ruixiong Deng:** data curation (equal), formal analysis (equal), investigation (equal), software (equal), visualization (equal), writing – original draft (equal). **Kaitong Xiao:** investigation (equal), resources (equal), software (equal), validation (equal), visualization (equal). **Xin Chen:** investigation (equal), resources (equal), software (equal), supervision (equal), visualization (equal). **Beibei Huang:** investigation (equal), visualization (equal). **Haoran Li:** investigation (equal), supervision (equal). **Lin Wu:** supervision (equal), writing – review and editing (equal). **Hang Ning:** conceptualization (equal), funding acquisition (equal), investigation (equal), methodology (equal), project administration (equal), resources (equal), supervision (equal), writing – review and editing (equal). **Hui Chen:** project administration (equal), supervision (equal), writing – review and editing (equal).

## Conflicts of Interest

The authors declare no conflicts of interest.

## Supporting information


Data S1.

## Data Availability

All environmental variables used in the manuscript are already publicly accessible, and we have provided the download address in the manuscript; relevant sampling site information can be found in Table S1 in the online version. Data can be accessed via a private link for peer review at: https://osf.io/6ynuv/files/osfstorage.
