# Supplementary material for: Impact of Climate Change on the Narrow Endemic Herb Psilopeganum sinense (Rutaceae) in China
Source: Ecol Evol. 2025 Feb 28;15(3):e71042. doi: 10.1002/ece3.71042 (PMC11868837; doi:10.1002/ece3.71042)
Supplement: Supplementary file 1 — Data S1. [file ECE3-15-e71042-s001.docx]

**Table S1**

**TableS1.** Coordinates of *Psilopeganum sinense*

|  | Species | Longitude (°) | Latitude (°) | Altitude (m) |
| --- | --- | --- | --- | --- |
| 1 | *Psilopeganum sinense* | 110.90 | 31.20 | 1001 |
| 2 | *Psilopeganum sinense* | 111.30 | 31.00 | 643 |
| 3 | *Psilopeganum sinense* | 107.80 | 30.00 | 358 |
| 4 | *Psilopeganum sinense* | 107.00 | 30.80 | 270 |
| 5 | *Psilopeganum sinense* | 109.50 | 31.00 | 302 |
| 6 | *Psilopeganum sinense* | 107.80 | 29.30 | 346 |
| 7 | *Psilopeganum sinense* | 109.50 | 30.00 | 768 |
| 8 | *Psilopeganum sinense* | 106.30 | 29.30 | 299 |
| 9 | *Psilopeganum sinense* | 110.80 | 31.30 | 776 |
| 10 | *Psilopeganum sinense* | 110.30 | 31.00 | 630 |
| 11 | *Psilopeganum sinense* | 109.60 | 31.40 | 453 |
| 12 | *Psilopeganum sinense* | 106.40 | 29.80 | 330 |
| 13 | *Psilopeganum sinense* | 106.60 | 29.50 | 428 |
| 14 | *Psilopeganum sinense* | 110.80 | 31.40 | 955 |
| 15 | *Psilopeganum sinense* | 109.90 | 31.10 | 294 |
| 16 | *Psilopeganum sinense* | 111.28 | 31.09 | 817 |
| 17 | *Psilopeganum sinense* | 111.12 | 31.00 | 780 |
| 18 | *Psilopeganum sinense* | 110.99 | 31.03 | 804 |
| 19 | *Psilopeganum sinense* | 111.24 | 31.05 | 710 |
| 20 | *Psilopeganum sinense* | 111.27 | 31.10 | 817 |
| 21 | *Psilopeganum sinense* | 111.11 | 30.98 | 780 |
| 22 | *Psilopeganum sinense* | 110.73 | 31.09 | 809 |
| 23 | *Psilopeganum sinense* | 110.86 | 31.21 | 754 |
| 24 | *Psilopeganum sinense* | 110.74 | 31.41 | 804 |
| 25 | *Psilopeganum sinense* | 110.70 | 31.17 | 820 |
| 26 | *Psilopeganum sinense* | 110.60 | 31.37 | 823 |
| 27 | *Psilopeganum sinense* | 110.82 | 31.35 | 822 |
| 28 | *Psilopeganum sinense* | 110.27 | 31.11 | 767 |
| 29 | *Psilopeganum sinense* | 110.37 | 30.94 | 804 |
| 30 | *Psilopeganum sinense* | 110.10 | 31.09 | 848 |
| 31 | *Psilopeganum sinense* | 110.47 | 31.21 | 696 |
| 32 | *Psilopeganum sinense* | 110.30 | 31.33 | 831 |
| 33 | *Psilopeganum sinense* | 109.86 | 32.19 | 721 |
| 34 | *Psilopeganum sinense* | 110.17 | 32.44 | 733 |
| 35 | *Psilopeganum sinense* | 109.81 | 30.61 | 795 |
| 36 | *Psilopeganum sinense* | 109.87 | 32.22 | 780 |
| 37 | *Psilopeganum sinense* | 109.34 | 31.18 | 841 |
| 38 | *Psilopeganum sinense* | 110.22 | 31.05 | 791 |
| 39 | *Psilopeganum sinense* | 109.53 | 31.30 | 875 |
| 40 | *Psilopeganum sinense* | 109.53 | 31.31 | 875 |
| 41 | *Psilopeganum sinense* | 109.40 | 31.08 | 822 |
| 42 | *Psilopeganum sinense* | 109.46 | 31.19 | 662 |
| 43 | *Psilopeganum sinense* | 110.10 | 31.72 | 802 |
| 44 | *Psilopeganum sinense* | 109.69 | 31.40 | 687 |
| 45 | *Psilopeganum sinense* | 109.98 | 30.96 | 720 |
| 46 | *Psilopeganum sinense* | 109.63 | 30.88 | 822 |
| 47 | *Psilopeganum sinense* | 110.04 | 31.95 | 798 |
| 48 | *Psilopeganum sinense* | 109.54 | 31.18 | 864 |
| 49 | *Psilopeganum sinense* | 107.82 | 30.64 | 747 |
| 50 | *Psilopeganum sinense* | 106.79 | 30.20 | 823 |
| 51 | *Psilopeganum sinense* | 107.53 | 30.65 | 759 |
| 52 | *Psilopeganum sinense* | 110.01 | 30.96 | 729 |
| 53 | *Psilopeganum sinense* | 109.30 | 31.21 | 801 |
| 54 | *Psilopeganum sinense* | 105.93 | 28.95 | 349 |
| 55 | *Psilopeganum sinense* | 106.13 | 28.98 | 438 |
| 56 | *Psilopeganum sinense* | 105.97 | 29.02 | 272 |
| 57 | *Psilopeganum sinense* | 107.07 | 29.50 | 674 |
| 58 | *Psilopeganum sinense* | 107.03 | 29.52 | 654 |
| 59 | *Psilopeganum sinense* | 108.67 | 31.22 | 670 |

**Table S2**

**Table S2.** 19 bioclimatic variables

| Abbreviation (Unit) | Parameter |
| --- | --- |
| BIO1 | Annual Mean Temperature (°C) |
| BIO2 | Mean Diurnal Range (Mean of monthly (max temp - min temp)) (°C) |
| BIO3 | Isothermality (BIO2/BIO7) (*100) (%) |
| BIO4 | Temperature Seasonality (standard deviation x100) (°C) |
| BIO5 | Max Temperature of Warmest Month (°C) |
| BIO6 | Min Temperature of Coldest Month (°C) |
| BIO7 | Temperature Annual Range (°C) |
| BIO8 | Mean Temperature of Wettest Quarter (°C) |
| BIO9 | Mean Temperature of Driest Quarter (°C) |
| BIO10 | Mean Temperature of Warmest Quarter (°C) |
| BIO11 | Mean Temperature of Coldest Quarter (°C) |
| BIO12 | Annual Precipitation (mm) |
| BIO13 | Precipitation of Wettest Month (mm) |
| BIO14 | Precipitation of Driest Month (mm) |
| BIO15 | Precipitation Seasonality (Coefficient of Variation: mean/SD*100) (%) |
| BIO16 | Precipitation of Wettest Quarter (mm) |
| BIO17 | Precipitation of Driest Quarter (mm) |
| BIO18 | Precipitation of Warmest Quarter (mm) |
| BIO19 | Precipitation of Coldest Quarter (mm) |

**Table S3**

**Table S3.** Model evaluation indicators

| Index | Extremely high | Very high | High | Average | Fail |
| --- | --- | --- | --- | --- | --- |
| AUC | 1.0-0.9 | 0.9-0.8 | 0.8-0.7 | 0.7-0.6 | 0.6-0.5 |
| Kappa | 1-0.85 | 0.85-0.74 | 0.74-0.65 | 0.65-0.5 | <0.5 |
| TSS | 1-0.81 | 0.81-0.74 | 0.74-0.61 | 0.61-0.5 | <0.5 |

**Figure S1**


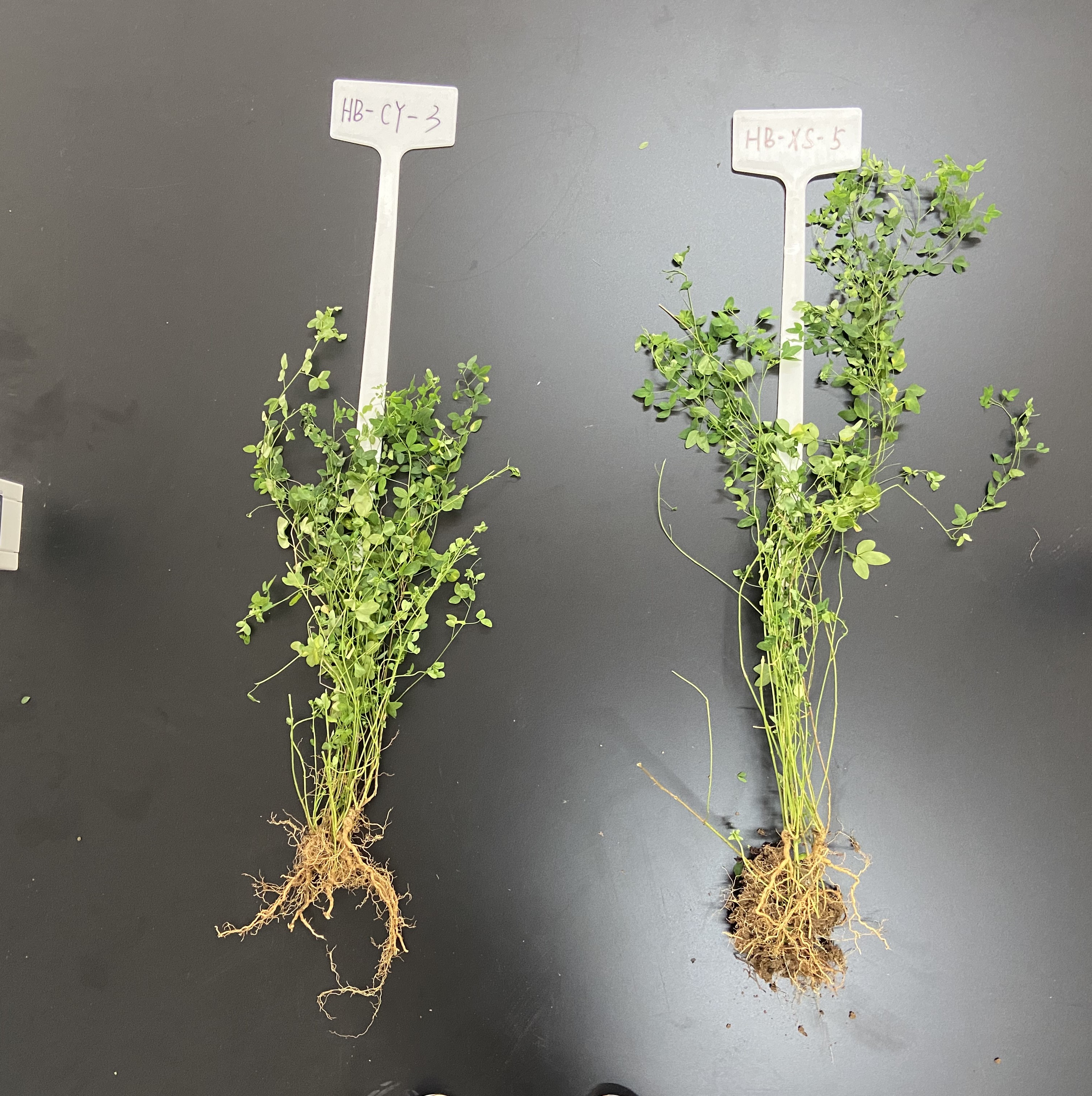

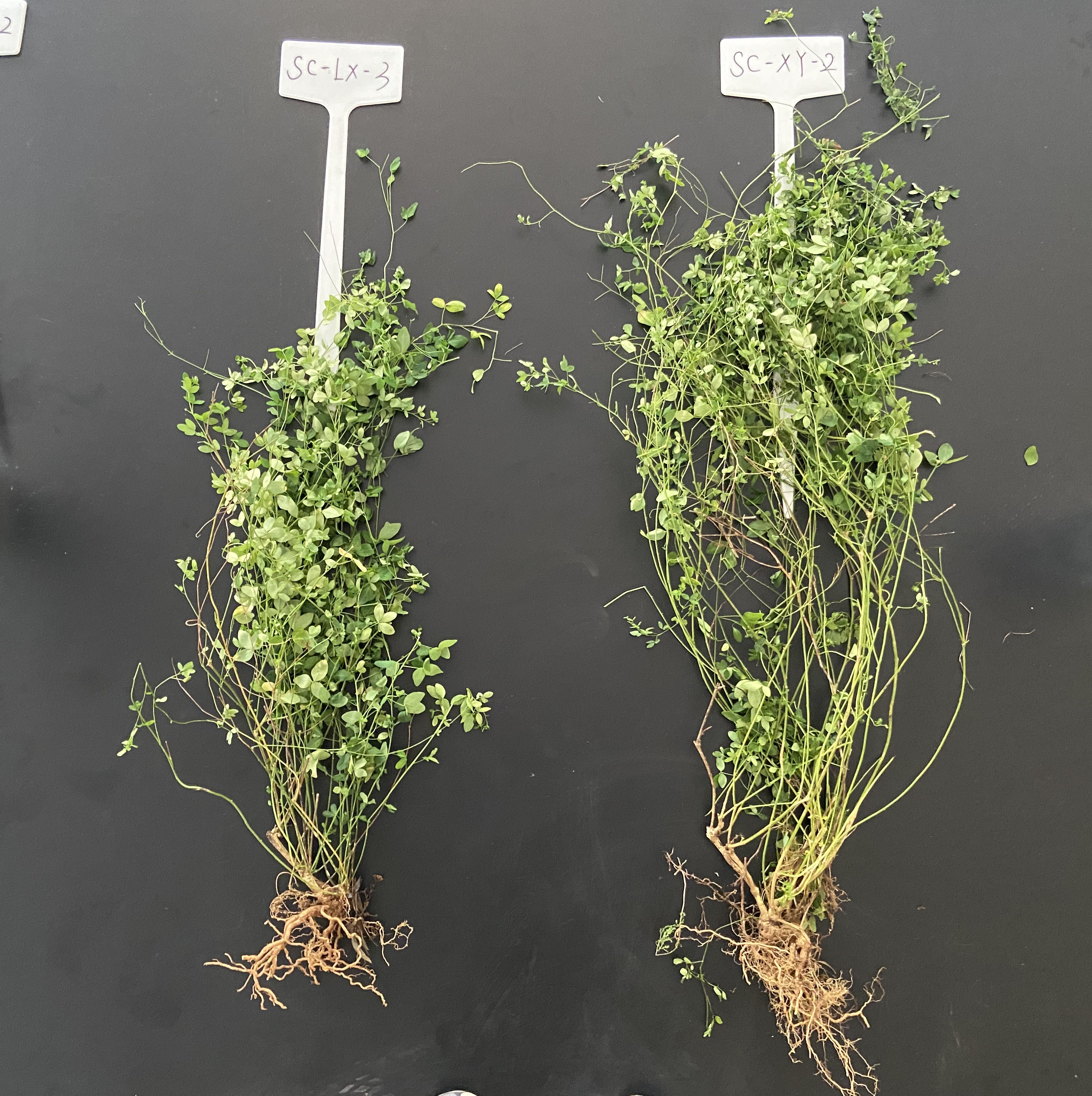


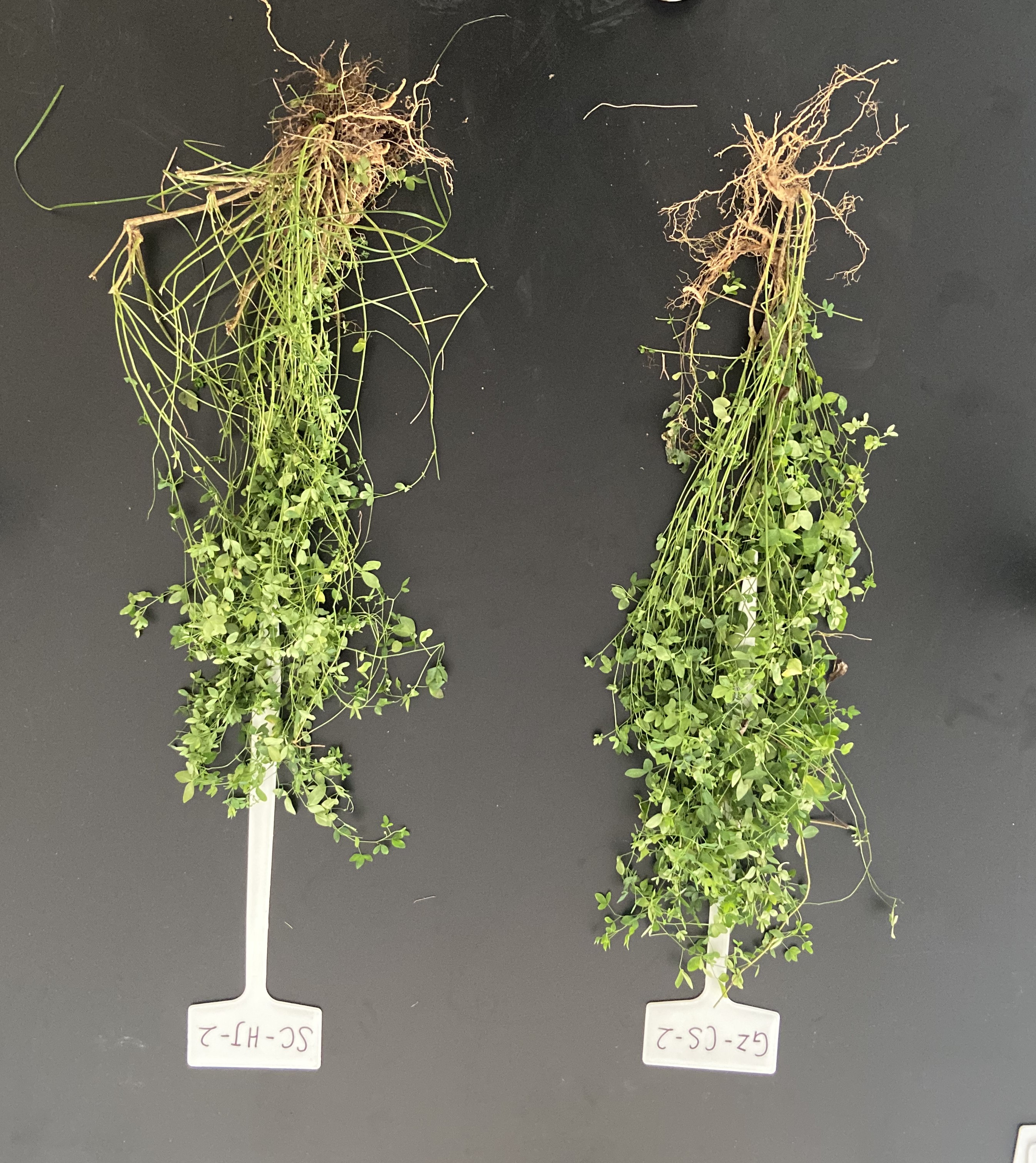

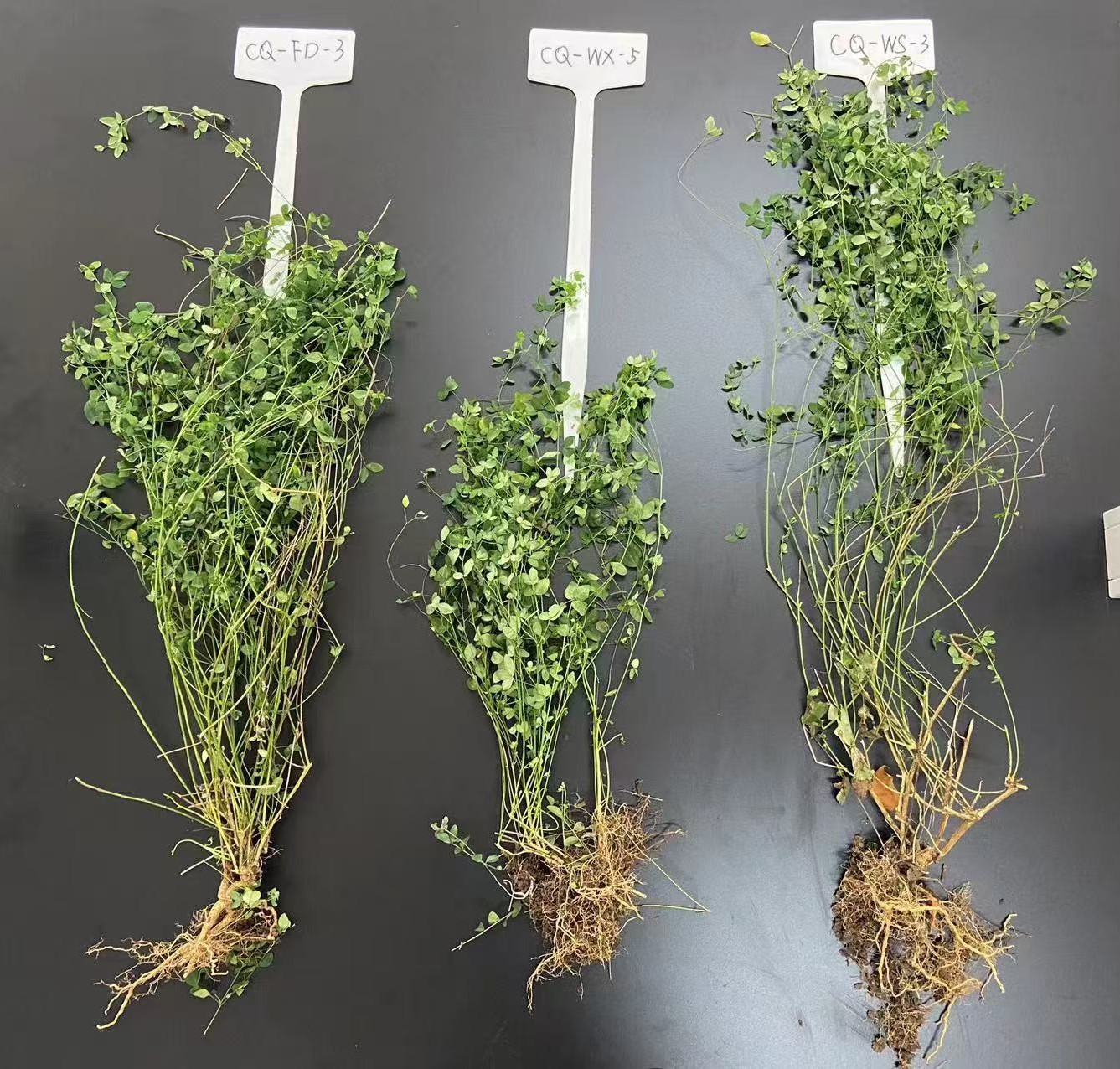


**Figure S1.** *Psilopeganum sinense* samples collected.
